# Associations between family factors and body weight gain from 20 years old

**DOI:** 10.1186/s12905-019-0719-0

**Published:** 2019-02-12

**Authors:** Wakako Suzuki, Wuren, Kiyonori Kuriki, Shizuoka-Sakuragaoka J-MICC Study Group

**Affiliations:** 1 School of Nursing, University of Shizuoka, 2-2-1 Oshika, Suruga-ku, Shizuoka, 422-8021 Japan; 2 Laboratory of Public Health, Graduate School of Integrated Pharmaceutical and Nutritional Sciences, University of Shizuoka, 52-1 Yada, Suruga-ku, Shizuoka, 422-8526 Japan

**Keywords:** Body weight gain, Obesity, Family structure, Family relationship, Marital status

## Abstract

**Background:**

Although family factors can greatly impact adult health, little is known about the extent to which family factors are related to body weight gain (BWG) in adulthood. This study aimed to examine the associations between family factors and BWG from 20 years old.

**Methods:**

Among the 6395 possible participants aged 35 to 79 years, 2884 men and 2171 women were eligible for the study. Present body mass indexes (BMI) were measured, and family factors and body weight from 20 years old (i.e., BMI_20yr) were collected using a self-administered questionnaire. The differences between BMI and BMI_20yr were calculated, and those with increases of BMI ≥2.5 kg/m^2^ (i.e., ≥7.5 and 6.0 kg in men and women, respectively) were defined as ‘cases’ of BWG. Using a multiple logistic regression analysis, the odds ratios (ORs, 95% confidence intervals [CIs] and p for trend) were estimated.

**Results:**

In the men, no association was found. In the women, the ORs were 0.31, 1.00 and 0.77 (0.17–0.58, [reference], and (0.52–1.29), *p* < 0.001) as per their marital status: unmarried, married, and bereaved/divorced, respectively. Although no association was found with family structure (i.e., single, couple, and two and three generations living together), for familial relationships, the ORs were 1.00, 1.11 and 1.86 ([reference], 0.85–1.46, and 1.25–2.79, *p* < 0.01) for ‘good’, ‘somewhat good’, and ‘not so good/not good’, respectively. Even if a ‘case’ of BWG was ≥3.5 kg/m^2^, nearly the same risks remained.

**Conclusion:**

Marital status and family relationships were associated with decreased and increased risks of BWG only in the female participants. Family factors should be considered when advising women on body weight control.

## Background

Obesity is an important public health concern world-wide, and adulthood body weight gain (BWG) is suggested to be related to increased risks of death from many diseases such as myocardial infarction [1] and colorectal cancer [2]. According to annual reports by the National Nutrition Survey in Japan [3], the prevalence rates of obesity among middle-aged to older men and women have consistently increased and remained at a higher level over the last 10 years. Certainly, the rate for young people has increased, and a remarkable BWG is also observed in adulthood, which has been thought to be particularly influenced by family contexts [4].

For children and young people, BWG has been reported to be positively related to perinatal, familial, and socio-economic factors [5–7]. Whereas, for adults, family structure, i.e., alone, couple, two and three generations, has been thought to be associated with being overweight and obese [8], but the details have not been clarified. Among paired adult siblings in a twin study, if one sibling became obese, it has been suggested that the other would also become obese [9]. As for the reasons why this would occur, it was suggested that while their parent’s contributions had an effect on cumulative BWG process of over the long term, lifestyle factors might be modified with adulthood BWG.

Gender differences were observed in the acceleration of BWG in adulthood [10]. In contrast to married American men, among married American women, BWG has been reported to continue during their lifetimes, but weight loss was observed only in women who were bereaved/divorced (i.e., widow) [11]. In a large-scale study among Finnish men and women, BWG was found with married people in a six-year follow-up, but weight loss was found with widows [12]. Among children and adolescents [13], increased risks of being overweight and obese have been shown to be associated with poor family relationships such as difficulty in communicating their parents, while little is known about their parents themselves. In East Asia, the divorce and single rates have gradually increased [14], so it is also considered to be a social issue.

As described above, although it has been suggested that living with one’s family may influence BWG [15], as of yet, family factors have not been studied regarding BWG. In the present cross-sectional study as part of the Shizuoka-Sakuragaoka Japan Multi-Institutional Collaborative Cohort (J-MICC) Study on adulthood BWG, we examined associations with family factors, which focused on family structure, marital status, and family relationships in each gender, and numbers of pregnancies, childbirth, and miscarriages with women.

## Methods

### Study participants

The present cross-sectional study was conducted as a series of the Shizuoka-Sakuragaoka J-MICC Study, which is summarized elsewhere [16]. In brief, the J-MICC study was executed to clarify relationships between genetic and lifestyle factors for risks of lifestyle-related diseases, such as cancer, heart and cerebrovascular diseases, and diabetes. The J-MICC Study was composed of 12 regional groups across all of Japan. More than 100 thousand baseline study participants were recruited between 2005 and 2013. The J-MICC Study and each regional study are ongoing according to common and regional protocols (which include all common protocols).

In the Shizuoka-Sakuragaoka J-MICC Study, the individuals who met the following criteria were recruited: 1) men and women aged 35 to 79 years; 2) inhabitants of our target regions (14 cities and three towns, from central to western locations in Shizuoka prefecture, where almost 1.7 million people are aged 35 to 79 years old, and sub-prefecture covers 5733 km^2^ out of the prefectural total 7777 km^2^); and 3) people who scheduled their annual health check-ups at our five collaborating institutes. We obtained individually written informed consents after thoroughly explaining the study’s purpose and outline with an explanatory document mailed beforehand and by trained research nurses. Along with Nagoya University Graduate School of Medicine and Aichi Cancer Center Research Institute, our study was approved by the Ethics Committee of University of Shizuoka (No. 22–39) and the collaborating health check-up centers. Over three years starting from February 2011, 6395 inhabitants in the target area participated, and participation rate was 27.6% for almost all of the corresponding health check-up examiners.

In the present study, 2884 men and 2171 women were finally selected as eligible participants after excluding for the following criteria: 1) missing data on family factors, body weight at 20 years old, lifestyle information used as potential confounding factors and measured height and body weight (*n* = 830), and 2) participants with their own medical history of cancer, cardiovascular disorders, cerebrovascular diseases, or diabetes mellitus (*n* = 839).

### Data collection on lifestyle information and family factors

Our self-administered questionnaire was composed of both common (e.g., current height and body weight, body weight at 20 years old, conventional lifestyle, and their own medical history) and original parts from all Japan and our region. In the former, number of pregnancies, childbirth, and miscarriages were included. In the latter, the following questions were listed: family factors such as marital status (i.e., unmarried, married, bereaved or divorce), family structure based on those living in the same house (i.e., single, alone due for job purposes, couple, two generations or three generations), family relationships between family members (i.e., good, somewhat good, ‘not so good’ or ‘not good’), and others. Along with the participants, trained scientific nurses reviewed their answered questionnaires to as much as possible limit missing data; this review was performed two to three times by other trained scientific nurses, using blue- and red-inked pens, respectively.

### Definition and cases of BWG

Anthropometric factors such as height and body weight were measured by making participants wear a light gown, and their body mass index (BMI) was subsequently calculated. The BMI at 20 years (BMI_20yr) was also calculated based on data in their questionnaires. Regarding the validity of recall bias for BMI_20yr, in a study among participants aged 50 (±2.2) years, the differences between long-term self-reported weight and actual measured weight at age 18 were about − 2.0 to + 2.0 kg and − 2.0 to − 1.5 kg in men and women, respectively [17]. Underestimation was observed in the women. Even among the male and female participants aged > 70 (< 1.0 as an absolute value of SD) years, their self-reported body weight 28 years ago was reported within the differences of 1.5 to 2.5 kg [18]. Similar findings were observed among Japanese men [19].

In Japan, BMI ≥25 kg/m^2^ has been defined as obese according to the Japan Society for the Study of Obesity, as per the WHO’s expert observation [20]. With reference to a previous study [21], an increment of BMI ≥2.5 kg/m^2^ from BMI_20yr was defined as a ‘case’ of BWG (Model 1), which meant a BWG ≥7.5 and 6.0 kg among Japanese men and women of average height, respectively. An increase of BMI ≥3.5 kg/m^2^ from BMI_20yr was also defined as a ‘case’ of BWG (Model 2), which corresponded with a BWG ≥10.0 and 8.4 kg, respectively. Each ‘negative’ BWG, that is, < 2.5 and < 3.5 kg/m^2^ from BMI_20yr in Models 1 and 2, respectively, were defined as the corresponding ‘control’. Thus, in the current study, the two models for analysing the risks of BWG ≥2.5 and ≥ 3.5 kg/m^2^ were set for the following reason: the differences in weight between Models 1 and 2 were almost 2.5 kg/m^2^ in each gender, and those were slightly larger than the recall bias for body weight at 20 years of age. Considering under- and overestimation of body weight at 20 years of age, it was checked whether the two risks were consistent by means of the two models in each gender.

Finally, to estimate the risks in the two models, there were 1125 and 673 cases of BWG in men, and 706 and 482 cases of BWG in women in Models 1 and 2, respectively.

### Statistical analyses

All analyses were performed separately for men and women. Regarding health consciousness with respect to body weight at baseline, but not at 20 years of age, partial Pearson’s correlation coefficients (r) were calculated between the two current BMI values according to actual measurements and the self-administered questionnaire, and the values were 0.98 and 0.99 (*p* < 0.001 for both) for men and women, respectively. Due to the small numbers of participants, the following levels were totaled as one level: ‘bereavement/divorce’ for marital status, ‘single/alone due to job purpose’ (labelled as ‘single’) for family structure, and ‘not so good/not good’ for family relationships. As appropriate, t- and chi-square tests were used for continuous and qualitative variables, respectively. In Models 1 and 2, a multiple logistic regression analysis was used to estimate the odds ratios (ORs) and 95% confidence intervals (CIs) for BMI increments of ≥2.5 and 3.5 kg/m^2^ from BMI_20yr, respectively. With reference to previous studies [8, 11, 12], the potential confounding factors were applied as follows: age, BMI, and physical activity (as continuous variables for the three variables), smoking status, and habitual drinking (never, ex- and smokers/drinkers = 0, 1 and 2), feeling stressed (many times, normal and rare = − 1, 0 and 1) and education level (< 12, 12 and ≥ 12 years = 0, 1 and 2). Trend association was assessed through the following assignments: ordinal numbers (− 1, 0 and 1) to unmarried, married and ‘bereavement/divorce’ for marital status; those (0, 1, 2 and 3) to single, couple, two generations and three generations for family structure; those (0, 1 and 2) to the following four variables for family relationships: good, somewhat good and ‘not so good/not good’ and for 0, 1 or 2 times of pregnancies, childbirth and miscarriages. A *p*-value of < 0.05 (two tails) was considered statistically significant. Statistical analyses were performed with the software packages SPSS version 19.0 (IBM Co., Armonk, NY).

## Results

Table 1 shows study participants’ characteristics according to two BWG models for the men: ≥2.5 and 3.5 kg/m^2^. In Model 1 (BWG ≥2.5 kg/m^2^), mean values and SD of age and BMI in male cases were younger and higher (56.0 ± 9.8 vs. 57.6 ± 11.1 years old, and 25.3 ± 2.8 vs. 21.9 ± 2.4 kg/m^2^, *p* < 0.001 for both), while BMI_20yr was lower (20.9 ± 2.2 vs. 21.7 ± 2.4 kg/m^2^, *p* < 0.001). Likewise, in Model 2 (BWG ≥ 3.5 kg/m^2^), the mean values and SD of age and BMI were younger and higher (56.1 ± 9.5 vs. 57.3 ± 11.0 years old, and 26.1 ± 2.9 vs. 22.4 ± 2.5 kg/m^2^, *p* = 0.01 and < 0.001, respectively), and BMI_20yr was lower (20.7 ± 2.3 vs. 21.6 ± 2.4 kg/m^2^, *p* < 0.001). In Models 1 and 2, the prevalence rates of obesity were 48.4 and 61.5% for male cases and 9.0 and 13.1% for male controls (*p* < 0.001 and 0.013), respectively.

**Table 1:** Characteristics of male study participants (*n* = 2884)

	Model 1 (⊿BMI> = 2.5 kg/m^2^)^a^			Model 2 (⊿BMI> = 3.5 kg/m^2^)^a^		
	Cases^b, c^	Contorls^b, c^	*p* ^c^	Cases^b, c^	Contorls^b, c^	*p* ^d^
	(*n* = 1125)	(*n* = 1759)		(*n* = 673)	(*n* = 2211)	
Age (yr)^e^	56.0 ± 9.8	57.6 ± 11.1	<0.001	56.1 ± 9.5	57.3 ± 11.0	<0.05
BMI (kg/m^2^)^e^	25.3 ± 2.8	21.9 ± 2.4	<0.001	26.1 ± 2.9	22.4 ± 2.5	<0.001
Height (cm)^e^	168.8 ± 5.8	168.1 ± 6.4	<0.05	168.9 ± 5.9	168.3 ± 6.3	<0.05
Weight (kg)^e^	72.4 ± 9.5	62.9 ± 8.3	<0.001	74.6 ± 9.7	64.2 ± 8.6	<0.001
Obesity, n (%)	545 (48.4)	159 (9.0)	<0.001	414 (61.5)	290 (13.1)	<0.05
BMI on 20 yr. (kg/m^2^)^e^	20.9 ± 2.2	21.7 ± 2.4	<0.001	20.7 ± 2.3	21.6 ± 2.3	<0.001
Weight on 20 yr. (kg)^e^	59.5 ± 7.7	61.3 ± 8.1	<0.001	59.2 ± 7.7	61.1 ± 8.0	<0.001
Obesity, n (%)	52 (4.6)	141 (8.0)	<0.001	31 (4.6)	162 (7.3)	<0.001
Physical activity (METs)^e^	18.6 ± 13.7	21.7 ± 14.3	<0.001	17.6 ± 13.5	21.3 ± 14.2	<0.001
Smoking status, n (%)	1122	1758	<0.001	671	2209	<0.05
Never smokers	326 (29.1)	591 (33.6)		197 (29.4)	720 (32.6)	
Former smokers	530 (47.3)	698 (39.7)		320 (47.7)	908 (41.1)	
Current smokers	266 (23.8)	469 (26.7)		154 (23.0)	581 (26.3)	
Habitual drinking, n (%)	1125	1759	0.057	673	2211	<0.05
Never drinkers	270 (24.0)	356 (20.2)		168 (25.0)	458 (20.7)	
Former drinkers	17 (1.5)	28 (1.6)		6 (0.9)	39 (1.8)	
Current drinkers	838 (74.5)	1375 (78.2)		499 (74.1)	1714 (77.5)	
Stress Feeling, n (%)	1051	1673	0.081	631	2093	0.538
Many times	156 (13.9)	252 (14.3)		92 (13.7)	316 (14.3)	
Normal	565 (50.2)	947 (53.8)		341 (50.7)	1171 (53.0)	
Nothing	330 (35.9)	474 (31.8)		198 (35.7)	606 (32.7)	
Education, n (%)	1122	1754	<0.05	672	2204	<0.05
< 12 yr	77 (6.9)	159 (9.0)		46 (6.8)	190 (8.6)	
12 yr	475 (42.3)	788 (44.8)		292 (43.5)	971 (44.1)	
> = 12 yr	570 (50.8)	807 (46.2)		334 (49.7)	1043 (47.3)	
Marital status, n (%)	1125	1759	0.352	673	2211	0.122
Unmarried	91 (8.1)	119 (6.8)		59 (8.8)	151 (6.8)	
Married	970 (86.2)	1547 (87.9)		572 (85.0)	1945 (88.0)	
Bereavement/divorce	64 (5.7)	93 (5.3)		42 (6.2)	115 (5.2)	
Family structure, n (%)	1121	1758	0.882	671	2208	0.164
Single	68 (6.1)	96 (5.5)		49 (7.3)	115 (5.2)	
Couple	218 (19.4)	355 (20.2)		123 (18.3)	450 (20.4)	
Two generations	519 (46.3)	817 (46.4)		309 (46.1)	1027 (46.5)	
Three generations	316 (28.2)	490 (27.9)		190 (28.3)	616 (27.9)	
Family relationships, n (%)	1121	1750	0.577	670	2201	0.542
Good	505 (44.9)	762 (43.3)		305 (45.5)	962 (43.7)	
Somewhat good	607 (54.0)	969 (55.1)		357 (53.0)	1219 (55.4)	
‘Not so good’/‘not good’	9 (0.8)	19 (1.1)		8 (1.2)	20 (0.9)	

*BMI* body mass index

^a^Increment of BMI ≥2.5 and 3.5 kg/m^2^ from BMI_20yr were meant ≥7.5 and 10.0 kg of body weight gain among Japanese men with their average height, in Model 1 and 2, respectively

^b^Cases and controls were defined as the study participants with ‘positive’ and ‘negative’ of each cut-off value on body weight gain in Model 1 and 2, respectively

^c^Numbers of ‘unknown’ were not shown

^d^t- or chi-square tests

^e^Values were shown as mean ± standard deviation

Among the women, Table 2 shows study participants’ characteristics according to two BWG models: ≥2.5 and 3.5 kg/m^2^. In contrast to the men, in Model 1, no differences were found between the two groups for age and BMI, but female cases had lower BMI_20yr (20.2 ± 2.3 vs. 20.9 ± 2.3 kg/m^2^, *p* < 0.001). In Model 2, they had higher BMIs and lower BMI_20yr (25.9 ± 3.5 vs. 20.8 ± 2.4, and 20.3 ± 2.4 vs. 20.8 ± 2.3 kg/m^2^, *p* < 0.001 for both, respectively). In Models 1 and 2, the prevalence rates of obesity were 43.5 and 55.2% for female cases and 3.0 and 5.0% for female controls (p < 0.001 for both), respectively.

**Table 2:** Characteristics of female study participants (*n* = 2171)

	Model 1 (⊿BMI> = 2.5 kg/m^2^)^a^			Model 2 (⊿BMI> = 3.5 kg/m^2^)^a^		
	Cases^b, c^	Contorls^b, c^	*p* ^c^	Cases^b, c^	Contorls^b, c^	*p* ^d^
	(n = 706)	(n = 1465)		(n = 482)	(n = 1689)	
Age (yr)^e^	54.8 ± 9.7	54.6 ± 10.8	0.651	54.7 ± 9.7	54.7 ± 10.7	0.972
BMI (kg/m^2^)^e^	25.0 ± 3.4	20.5 ± 2.3	0.123	25.9 ± 3.5	20.8 ± 2.4	<0.001
Height (cm)^e^	155.6 ± 5.3	155.7 ± 5.8	0.259	155.7 ± 5.4	155.6 ± 5.7	0.715
Weight (kg)^e^	60.6 ± 8.9	49.7 ± 6.1	<0.001	63.0 ± 9.1	50.4 ± 6.3	<0.001
Obesity, n (%)	307 (43.5)	44 (3.0)	<0.001	266 (55.2)	85 (5.0)	<0.001
BMI on 20 yr. (kg/m^2^)^e^	20.2 ± 2.3	20.9 ± 2.3	<0.001	20.3 ± 2.4	20.8 ± 2.3	<0.001
Weight on 20 yr. (kg)^e^	48.9 ± 6.1	50.5 ± 5.9	<0.001	49.2 ± 6.3	50.2 ± 5.9	<0.001
Obesity, n (%)	26 (3.7)	67 (4.6)	0.337	21 (4.4)	72 (4.3)	0.899
Physical activity (METs)^e^	31.4 ± 7.3	32.0 ± 7.3	0.075	31.3 ± 7.5	32.0 ± 7.3	0.076
Smoking status, n (%)	705	1463	<0.01	481	1687	<0.001
Never smokers	586 (83.0)	1282 (87.5)		394 (81.7)	1474 (87.4)	
Former smokers	73 (10.3)	97 (6.6)		57 (11.8)	113 (6.7)	
Current smokers	46 (6.5)	84 (5.7)		30 (6.2)	100 (5.9)	
Habitual drinking, n (%)	706	1464	0.393	482	1688	0.101
Never drinkers	411 (58.2)	832 (56.8)		290 (60.2)	953 (56.5)	
Former drinkers	13 (1.8)	18 (1.2)		10 (2.1)	21 (1.2)	
Current drinkers	282 (39.9)	614 (41.9)		182 (37.8)	714 (42.3)	
Stress Feeling, n (%)	706	1465	<0.01	482	1689	0.126
Many times	48 (6.8)	113 (7.7)		33 (6.8)	128 (7.6)	
Normal	300 (42.5)	713 (48.7)		208 (43.2)	805 (47.7)	
Nothing	358 (50.7)	639 (43.6)		241 (50.0)	756 (44.8)	
Education, n (%)	706	1465	0.139	482	1689	0.865
< 12 yr	51 (7.2)	98 (6.7)		33 (6.8)	116 (6.9)	
12 yr	356 (50.4)	752 (51.3)		247 (51.2)	861 (51.0)	
> = 12 yr	299 (42.4)	615 (42.0)		202 (41.9)	712 (42.2)	
Marital status, n (%)	706	1465	<0.001	482	1689	<0.05
Unmarried	23 (3.3)	107 (7.3)		14 (2.9)	116 (6.9)	
Married	607 (86.0)	1163 (79.4)		414 (85.9)	1356 (80.3)	
Bereavement/divorce	76 (10.8)	195 (13.3)		54 (11.2)	217 (12.8)	
Family structure, n (%)	705	1461	0.176	482	1684	0.266
Single	29 (4.1)	83 (5.7)		19 (16.8)	93 (19.1)	
Couple	120 (17.0)	282 (19.2)		81 (3.9)	321 (5.5)	
Two generations	358 (50.7)	688 (47.0)		247 (51.2)	799 (47.4)	
Three generations	198 (28.0)	408 (27.8)		135 (28.0)	471 (28.0)	
Family relationships, n (%)	703	1459	<0.001	480	1682	0.163
Good	273 (38.8)	642 (44.0)		187 (38.8)	728 (43.3)	
Somewhat good	339 (48.2)	674 (46.2)		233 (48.3)	780 (46.4)	
‘Not so good’/‘not good’	91 (12.9)	143 (9.8)		60 (12.4)	174 (10.3)	
Pregnancy, n (%)	705	1459	<0.001	482	1682	<0.001
Nothing	44 (6.2)	165 (11.3)		25 (5.2)	184 (10.9)	
1 time	61 (8.7)	100 (6.9)		46 (9.5)	115 (6.8)	
> =2 times	600 (85.1)	1194 (81.8)		411 (85.3)	1383 (82.2)	
Childbirth, n (%)	706	1465	<0.001	482	1689	<0.001
Nothing	56 (7.9)	195 (13.3)		34 (7.1)	217 (12.8)	
1 time	81 (11.5)	127 (8.7)		62 (12.9)	146 (8.6)	
> =2 times	569 (80.6)	1143 (78.0)		386 (80.1)	1326 (78.5)	
Miscarriage, n (%)	577	1122	0.557	391	1308	0.859
Nothing	483 (83.7)	950 (84.7)		333 (85.2)	1100 (84.1)	
1 time	62 (10.7)	123 (11.0)		41 (10.5)	144 (11.1)	
> =2 times	32 (5.5)	49 (4.4)		17 (4.3)	64 (4.9)	

*BMI* body mass index

^a^Increment of BMI ≥2.5 and 3.5 kg/m^2^ from BMI_20yr were meant ≥6.0 and 8.4 kg of body weight gain among Japanese women with their average height, in Model 1 and 2, respectively

^b^Cases and controls were defined as the study participants with ‘positive’ and ‘negative’ of each cut-off value on body weight gain in Model 1 and 2, respectively

^c^Numbers of ‘unknown’ were not shown

^d^t- or chi-square tests

^e^Values were shown as mean ± standard deviation

Tables 3 and 4 shows the multivariable-adjusted ORs (95% CIs) for BWG ≥2.5 and 3.5 kg/m^2^ (Models 1 and 2) according to family factors among the men and women, respectively. No association was found in the men. For the women, in Model 1 (Table 4), the ORs were 0.31, 1.00 and 0.77 (0.52–1.29, [reference] and 0.17–0.58, p for trend < 0.001) for marital statuses, that were ‘unmarried’, ‘married’, and ‘bereavement/divorce’, respectively. Further, the ORs were 1.00, 1.11 and 1.86 ([reference], 0.85–1.46 and 1.25–2.79, p for trend = 0.009) for familial relationships, that were ‘good’, ‘somewhat good’ and ‘not so good/not good’, respectively. For women, in Model 2, the corresponding two risks remained as follows: 0.89, 1.00 and 0.29 (0.58–1.39, [reference] and 0.13–0.65, p for trend = 0.006), and 1.00, 0.98 and 1.62 ([reference], 0.72–1.33 and 1.02–2.56, p for trend = 0.120), respectively.

**Table 3:** Multivariate logistic regression analyses of family factors for body weight gain in men

	Model 1 (⊿BMI> = 2.5 kg/m^2^)			Model 2 (⊿BMI> = 3.5 kg/m^2^)		
	ORs^a^	95% CIs^a^	p for trend^b^	ORs^a^	95% CIs^a^	p for trend^b^
Marital status						
Unmarried	0.77	(0.52–1.14)		0.79	(0.51–1.22)	
Married	1.00	(Ref)		1.00	(Ref)	
Bereavement/Divorce	1.10	(0.74–1.65)	0.835	1.27	(0.82–1.98)	0.311
Family structure						
Single	1.03	(0.68–1.56)		1.37	(0.88–2.15)	
Couple	1.11	(0.86–1.43)		0.99	(0.74–1.32)	
Two generations	1.00	(Ref)		1.00	(Ref)	
Three generations	1.13	(0.90–1.41)	0.776	1.14	(0.89–1.48)	0.934
Family relationships						
Good	1.00	(Ref)		1.00	(Ref)	
Somewhat good	0.90	(0.75–1.09)		0.89	(0.72–1.10)	
‘Not so good’/‘Not good’	0.55	(0.19–1.54)	0.752	1.28	(0.45–3.66)	0.793

*CIs* confidence intervals, *ORs* odds ratios, *Ref* reference

^a^ORs and CIs were adjusted for age, BMI and physical activity (as continuous variables for the three variables), smoking status (never, ex- and smokers = 0, 1 and 2), habitual drinking (never, ex- and drinkers = 0, 1 and 2), feeling stressed (many times, normal and rare = − 1, 0 and 1) and education level (< 12, 12 and ≥ 12 = 0, 1 and 2)

^b^Trend association was assessed by assigning ordinal numbers (− 1, 0 and 1) to unmarried, married and ‘bereavement/divorce’ for marital status, those (0, 1, 2 and 3) to single, couple, two generations and three generations for family structure, and then those (0, 1 and 2) to good, somewhat good and “not so good/not good” for family relationships, respectively

**Table 4:** Multivariate logistic regression analyses of family factors for body weight gain in women

	Model 1 (⊿BMI> = 2.5 kg/m^2^)			Model 2 (⊿BMI> = 3.5 kg/m^2^)		
	ORs^a^	95% CIs^a^	p for trend^b^	ORs^a^	95% CIs^a^	p for trend^b^
Marital status						
Unmarried	0.31	(0.17–0.58)		0.29	(0.13–0.65)	
Married	1.00	(Ref)		1.00	(Ref)	
Bereavement/Divorce	0.77	(0.52–1.29)	< 0.001	0.89	(0.58–1.39)	0.006
Family structure						
Single	0.81	(0.46–1.42)		0.87	(0.45–1.69)	
Couple	1.37	(0.75–2.51)		1.18	(0.80–1.74)	
Two generations	1.00	(Ref)		1.00	(Ref)	
Three generations	1.06	(0.59–1.91)	0.445	0.82	(0.59–1.15)	0.224
Family relationships						
Good	1.00	(Ref)		1.00	(Ref)	
Somewhat good	1.11	(0.85–1.46)		0.98	(0.72–1.33)	
‘Not so good’/‘Not good’	1.86	(1.25–2.79)	0.009	1.62	(1.02–2.56)	0.120
Pregnancy						
Nothing	0.46	(0.29–0.74)		0.38	(0.21–0.68)	
1 time	0.99	(0.61–1.60)		1.06	(0.62–1.82)	
> = 2 times	1.00	(Ref)	0.003	1.00	(Ref)	0.004
Childbirth						
Nothing	0.45	(0.29–0.70)		0.40	(0.23–0.68)	
1 time	1.28	(0.84–1.95)		1.53	(0.96–2.43)	
> = 2 times	1.00	(Ref)	0.004	1.00	(Ref)	0.014
Miscarriage						
Nothing	1.00	(Ref)		1.00	(Ref)	
1 time	1.05	(0.68–1.61)		1.03	(0.64–1.68)	
> =2 times	1.30	(0.71–2.40)	0.429	0.70	(0.33–1.47)	0.490

*CIs* confidence intervals, *ORs* odds ratios, *Ref* reference

^a^ORs and CIs were adjusted for age, BMI and physical activity (as continuous variables for the three variables), smoking status (never, ex- and smokers = 0, 1 and 2), habitual drinking (never, ex- and drinkers = 0, 1 and 2), feeling stressed (many times, normal and rare = − 1, 0 and 1) and education level (< 12, 12 and ≥ 12 = 0, 1 and 2)

^b^Trend association was assessed by assigning ordinal numbers (− 1, 0 and 1) to unmarried, married and ‘bereavement/divorce’ for marital status, those (0, 1, 2 and 3) to single, couple, two generations and three generations for family structure, those (0, 1 and 2) to the following four variables: good, somewhat good and ‘not so good/not good’ for family relationships, and for 0, 1 or 2 times of pregnancies, childbirth and miscarriages, respectively

Moreover, regarding numbers of pregnancies and childbirth in Table 4, decreased risks were 0.46, 0.99, and 1.00 (0.29–0.73, 0.61–1.60 and [reference], p for trend = 0.003) and 0.45, 1.28, and 1.00 (0.29–0.70, 0.84–1.95 and [reference], p for trend = 0.004) for 0, 1 and 2 instances/people in Model 1, then 0.38, 1.06, and 1.00 (0.21–0.68, 0.62–1.82 and [reference], p for trend = 0.004) and 0.40, 1.53, and 1.00 (0.23–0.68, 0.96–2.43 and [reference], p for trend = 0.014), in Model 2, respectively.

## Discussion

The present study showed negative and positive associations with marital status and family relationships for BWG ≥2.5 kg/m^2^ at 20 years old, respectively, for women, but not men, and these associations remained for BWG ≥3.5 kg/m^2^ at 20 years old. Although we should note the under- and overestimation of recall bias for BWG from the age of 20 onwards, the relationships between family factors and BWG were consistent in the two models of BWG. In other words, with reference to ‘married’ and ‘good’, decreased and increased risks of significant BWG were found with the responses ‘bereavement/divorce’ and ‘not so good/not good’ among the women, respectively. Our findings with respective to the former are supported by a previous study [22]. For the latter, although not preferred, this study is the first report to show that BWG in adulthood (i.e., at least a ≥ 6.0 kg increase in weight among women with average height) was reduced because of poor relationships with their family members. Further, excepting miscarriages, pregnancies, and childbirth were also negatively related to BWG_20yr in the women.

With variables on family factors, marital status has been well-investigated with regards to the risks of becoming overweight and obese and BWG, especially after marriage or from the baseline survey. In contrast to previous studies, higher BWG in single women than in married women has been reported, and ‘marriage’ was also proposed to play in a role in suppressing BWG if women were the same age [23]. Among a large number of American women, no effect on marital BWG was observed in a 10-year follow-up [24]. In mothers (i.e., women who were raising their child/children), risks for being overweight and obese have been inconsistent [6]. Therefore, it is very important to investigate whether a women’s (wife’s and/or mother’s) weight increased due to marriage. When in their lifetime did they gain weight? Did body weight increase after marriage, but not during adolescence or young adulthood? Was BWG really caused by marriage [25]? How about family impacts? Especially for women, in the time that has elapsed since they were married, the following factors have also yet to be examined: pregnancy, childbirth, breast-feeding, child rearing, and menopause. BWG with some factors such as pregnancy and childbirth might be strongly related to and thus explained by marital status. Changes in the hormonal milieu caused by menopause would be associated with both increments in total body fat and abdominal fat [26].

In the present study, more than 80% of male and female participants were married, and divorce rates were lower in each gender. As with rates among Americans and Europeans, the divorce rates in Japanese were higher with young-middle aged couples, and lower with elderly-older ones, even if their wives (i.e., divorced women) could live on their own pensions after their husbands retired [27]. According to the National Nutritional Survey in Japan, BMI is higher among elderly-older women, whereas a higher proportion of young-middle aged women tend to advocate for being lean as public health issue [3]. Although the risk was adjusted for age as one of the confounding factors, appropriately, the effects of age might be not excluded for the risks of adulthood BWG in our statistical models. In many previous studies, however, ‘bereavement/divorce’ played in a role in suppressing BWG only among widowed/divorced women, but not their spouses [11, 12, 28]. In a follow-up study one year after marriage, widowed and divorced women had significant weight loss [11]. In a prospective cohort study, higher weight loss was observed between baseline and follow-up periods only in widows [28]. Many possible reasons were suggested: 1) their body weight was thought to be one of their essential female charm factors [29]; 2) women desire to maintain their body weight before and even after marriage [30]; 3) wives might be motivated to control their weight to maintain good relationships with their husbands [31]; 4) among married women, they might maintain their body shapes to have access to their cultural and social communities established before marriage [32].

In this study, we observed increased risks of adulthood BWG associated with poor family relationships only in women. Regarding family relationships between husbands and wives, responses for high level of distress have been observed at both extremes, i.e., weight loss in some individuals and weight gain in others [22]. Until today, enough evidence has not been accumulated. Women would be thought to have much familial frustration or distress with their family members at home. With reference to husbands/fathers, wives/mothers care for their husbands, children/their parents, and deal with their families for longer periods of time. Possibly, this may be supported by a previous study which reported on lifestyles in associations between obesity and eating behaviors, such as alcohol intake, long hours of television viewing, and lack of sleep [33]. The following lifestyles could be applied to men (as both husbands and fathers) with alcohol consumption (due to releasing their frustrations or distress), children and their parents with long hours of TV viewing, and women (as both wives and mothers) with insufficient sleep, respectively. Contrary to our expectations, in the present study, we failed to find associations between adulthood BWG and family structure. Also, no association was found with adulthood BWG in men, the reasons for which we cannot explain.

From the mental health viewpoint with women and families, ‘not so good/not good’ familial relationships are not proposed to reduce adulthood BWG. It would be complicated to distinct family relationships into marital status, such as psychological distress from family and stress from marital dissolution [34], in that order. Along with marital status, moreover, family relationships might be complicatedly associated with family structure. If so, risks of BWG with family relationships would be multiplicatively assessed. As one of the critical issues, answers to family relationships were subjectively, not quantitatively assessed, but it was quite difficult to appropriately evaluate them. With other critical issues, little has been known about associations between family relationships and BWG, and there might be gender and generational differences. Although the concept of family may be quite different between Asian and Western/American people, our findings would be available to help in the fight against excessive weight gain and obesity as world-wide public health concerns.

The present study has several limitations. First, the baseline data from the Shizuoka-Sakuragaoka J-MICC Study were used. Body weight at 20 years old was not measured, and was based on a non-validated self-administered questionnaire. The issue with the validity of self-reported BMI_20yr has been discussed in most previous epidemiological studies. Considering their health consciousness, we asked the participants to state their present height and weight, and subsequently checked the partial Pearson’s correlation coefficient between the two current BMI values. In our five-year follow-up research, we are now asking the participants the same questions again as a reproducibility test. Second, there could be a large systematic error in the differences between BMI_20yr and present BMI because of recall bias. However, as a critical public health issue, it is especially important to identify long-term risk factors for BWG over a long term, such as BMI_20yr. We have observed that excessive interpretation should be avoided for such findings. Third, most of the missing data might have been derived from obese participants, possibly obese women, which could contribute to selection bias. In the present study, all the participants were ≥ 35 years old, and most of them did not have their old health check-up data from at least 15 years prior. Fourth, the proportions of single and bereaved/divorce participants with respect to marital status were relatively small. Our sample size was nearly 6400, and family relationships would be available to estimate the risk of BWG. As with others, our study participants were recruited from only one prefecture in Japan, Shizuoka. Considering that this is a population-based study, the study participants were recruited by health check-up examiners, as the representative residents who live in 14 cities and three towns in Shizuoka Prefecture. The following variables were not adjusted for risks; householder status and individual income (data were not collected for both), years and the region from which the study participants were recruited (because the study was collected for a short period and in a limited area), and dietary intakes of foods and nutrients. Thus, further studies are needed to assess and identify the differences between the levels of family factors and their effects on the risks of BWG ≥ 2.5 and 3.5 kg/m^2^.

## Conclusions

In conclusion, we demonstrated decreased and increased risks of BWG ≥ 2.5 kg/m^2^ at 20 years old by marital status and family relationships (i.e., ‘bereavement/divorce’ and ‘not so good/not good’ vs. ‘married’ and ‘good’ as references) in the female participants, respectively, and that no association was observed in the male participants. These findings applied to BWG ≥3.5 kg/m^2^ at 20 years old and are supported by the results of previous studies among European/American people, especially with respect to marital status. It could be argued that BWG is suppressed by poor family relationships, and therefore further studies should investigate cause-effect relationships between BWG and family factors.

## Abbreviations

BMI: Body Mass Index
BMI_20yr: BMI from 20 years old
BWG: Body Weight Gain
CI: Confidence Interval
J-MICC Study: Japan Multi-Institutional Collaborative Cohort Study
OR: Odds Ratio
